# Chemical Compositions and Antibacterial Activities of the Essential Oils from Aerial Parts and Corollas of *Origanum acutidens* (Hand.-Mazz.) Ietswaart, an Endemic Species to Turkey

**DOI:** 10.3390/molecules14051702

**Published:** 2009-04-30

**Authors:** Belgin Cosge, Arzu Turker, Arif Ipek, Bilal Gurbuz, Neset Arslan

**Affiliations:** 1 Abant Izzet Baysal University, Mudurnu Vocational School of Higher Education, Mudurnu, Tr- 14800 Bolu, Turkey; 2 Abant Izzet Baysal University, Biology Department, Tr-14280 Bolu, Turkey; E-mail: turker_a@ibu.edu.tr (A.T.); 3 Ordu University, Faculty of Agriculture, Field Crops Department, Ordu, Turkey; E-mail: ipek@agri.ankara.edu.tr; 4 Ankara University, Faculty of Agriculture, Field Crops Department, 06110 Diskapi, Ankara, Turkey; E-mails: gurbuz@agri.ankara.edu.tr (B. G.); arslan@agri.ankara.edu.tr (N. A.)

**Keywords:** *Origanum acutidens* (Hand.-Mazz.), Essential oil, GC-MS analysis, Antibacterial activity.

## Abstract

Essential oils extracted by hydrodistillation from the aerial parts and corollas of *Origanum acutidens* (Hand.-Mazz.) Ietswaart, an endemic Turkish flora species, were analyzed by GC-MS. The amounts of essential oil obtained from the aerial parts and the corollas were 0.73% and 0.93%, respectively. Twenty-five components in both the aerial parts oil and the corolla oil, representing 95.11% and 93.88%, respectively, were identified. The aerial parts and corolla oils were characterized by the predominance of two components: *p*-cymene (9.43% and 17.51%) and carvacrol (67.51% and 52.33%), respectively. The essential oils were also evaluated for their antimicrobial activity against ten bacteria by the disc diffusion assay. Our findings showed the following order in the sensitivity to the essential oils, as indicated by the corresponding inhibition zones: *Proteus vulgaris > Salmonella typhimurium > Enterobacter cloacae > Klebsiella pneumonia > Escherichia coli > Serratia marcescens > Pseudomonas aeruginosa* for the aerial parts essential oil, and *Salmonella typhimurium > Proteus vulgaris > Enterobacter cloacae > Escherichia coli > Klebsiella pneumoniae > Serratia marcescens > Pseudomonas aeruginosa* for the corolla essential oil. The studied essential oils thus exhibited a broad-spectrum of activity against both Gram-positive and Gram-negative bacteria, whereas the tested Gram-positive bacteria were more susceptible to the essential oil samples.

## Introduction

Of the 250,000 species of flowering plants in the World, more than 20,000 – nearly 10 % of the total – are classified as herbs. Herbs picked by people from the wild have been an essential factor in health care all over the World throughout the ages and in all cultures. Nowadays, some 80 % of the World’s people rely on traditional, plant-based medicines for their primary health care. 

According to the most recent records, in Turkey there are 10,754 native plant species and 3,708 endemic plant species (an endemism ratio of about 34.8) [1]. The genus *Origanum* (belonging to the Lamiaceae) is represented by 22 species and four subspecies in Turkey, considered as the gene centre of this genus. They are grouped into eight sections and 14 species are endemic to Turkey. One such endemic species is *Origanum acutidens* (Hand.-Mazz.) Ietswaart. Some endemic *Origanum* species including *O. acutidens* (such as *O. bilgeri, O. hypercifolium,* and *O. sipyleum*) are used as herbal teas in the regions where they grow [2]. In addition, *Origanum* species are widely used as a culinary herb, to flavor food products and alcoholic beverages [3,4,5]. *O. acutidens* is a perennial herb of some 15-30 cm in height. It has gray-green fragrant leaves, yellow-green flower bracts and a white to pale yellow or pink corollas.

Essential oils have many applications in folk medicine and for flavoring and preservation, as well as in the fragrance and pharmaceutical industries. The antimicrobial property of essential oils has been known for a long time, and a number of investigations have been conducted on their antimicrobial activities, using bacteria, viruses and fungi as target organisms. 

A lot of *Origanum* plants are characterized by the existence of noticeable chemical differences with respect to both essential oil content and composition, and researchers have recently concentrated on these plants in particular, owing to the potential antimicrobial and antioxidant activities of their essential oils. The essential oil from the aerial parts of *Origanum acutidens* (Hand.-Mazz.) Ietswaart has been analyzed by several authors, and carvacrol and *p*-cymene were identified as the major components in this oil [6,7,8], and it was reported that the essential oil obtained from *Origanum acutidens* exhibited a significant antimicrobial activity against the bacteria used the study [7]. However, we have not found any published research on the content and composition of the essential oil from *Origanum acutidens* flowers. 

The aims of this study were to identify and compare the chemical composition of essential oils isolated by hydrodistillation from aerial parts and corollas of *Origanum acutidens* (Hand.-Mazz.) Ietswaart grown in Turkey by gas chromatography/mass spectrometry (GC/MS) analysis, and to evaluate the antimicrobial activities of these essential oils.

## Results and Discussion

The aerial parts and corolla gave dark yellowish and pale yellowish oils, respectively. The essential oil contents obtained were 0.73% in aerial parts and 0.93% in corollas. In previous studies [8,9,10] the essential oil obtained from the aerial parts of *O. acutidens* ranged from 0.6-1.4%. The essential oil components identified in the two samples are listed in Table 1, together with their relative percentages, in order of their retention indices. The qualitative compositions of aerial parts and corolla there were different. Twenty-five components in both aerial parts oil and corolla oil, representing 95.11% and 93.88%, respectively, were identified (Table 1).

**Table 1:** Chemical compositions (%) of essential oil in corolla and aerial parts of *O. acutidens.*

Peak no.	Compounds	RT (min)	Corolla (%)	Aerial Parts (%)
1	*α-*Phellandrene	9.58	0.55	-
2	*α -* Pinene	9.84	0.63	0.27
3	Camphene	10.45	0.73	0.31
4	2-*β*-Pinene	11.68	-	0.18
5	1-Octen-3-ol	11.85	-	0.74
6	3-Octanone	12.18	0.65	1.26
7	Myrcene	12.36	0.57	0.29
8	*α-*Terpinene	13.49	0.77	0.32
9	***p*-Cymene**	**13.89**	**17.51**	**9.43**
10	Eucalyptol	14.18	0.46	0.54
11	*γ-*Terpinene	15.47	0.79	0.44
12	*cis*-Sabinenehydrate	15.86	0.92	0.55
13	Linalol	17.44	0.70	-
14	*β-*Thujone	17.68	1.93	1.31
15	*α-* Thujone	18.21	-	0.31
16	Camphor	19.51	1.17	0.50
17	Isoborneol	20.49	2.21	1.36
18	Terpinene-4-ol	21.05	0.62	0.62
19	*p*-Cymen-8-ol	21.42	0.58	0.54
20	Dihydrocarvone	21.99	-	0.33
21	Tymoquinone	24.41	2.85	3.80
22	Linalyl butyrate	24.73	1.76	-
23	Borneol	26.12	1.19	-
24	Thymol	26.30	-	0.54
**25**	**Carvacrol**	**26.83**	**52.33**	**67.51**
26	*β-*Caryophyllene	31.79	2.04	1.62
27	*δ*-Cadinene	34.88	-	0.79
28	1H-Cycloprop[e]azulen-7-ol	38.14	-	0.46
29	Caryophyllene oxide	38.34	0.91	1.09
30	Isoelemicin	40.24	0.57	-
31	*S*-Indacene-1,7-dione	46.50	0.55	-
32	6-(2-Formylhydrazino)-N,N ^' ^–bis (isopropyl)-1,3,5-triazine-2,4-diamine	46.96	0.89	-
	**Amount of identified compounds**		**93.88**	**95.11**

**Figure 1:**
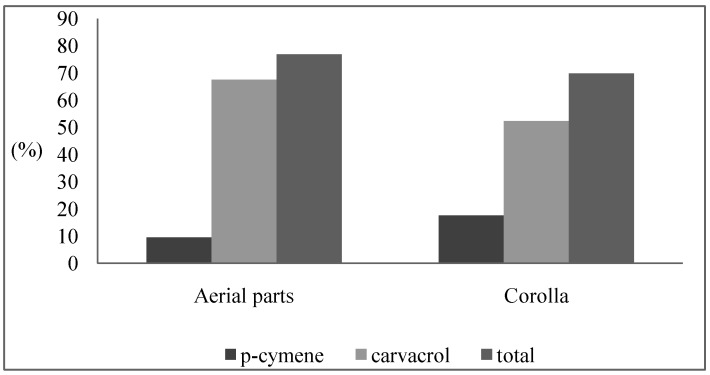
*p*-Cymene and carvacrol and their total relative amounts in the two essential oils (%).

In our study, *p*-cymene (9.43% and 17.51%) and carvacrol (67.51% and 52.33%) were found to be the major components of both the aerial parts and corolla oils, respectively. As seen Figure 1, the total of these two components were recorded higher in aerial parts (76.94%) than corolla (69.84%). Similarly to our findings, the contents of cavracrol and *p*-cymene measured in previous studies [6,8,10] varied from 65% to 87.0% and from 2.0% to 14.0%, respectively. 

The other important component of the aerial parts oil was tymoquinone (3.80%) This component (2.85%) was detected relatively low amount in corolla oil. *β-*thujone, camphor, isoborneol, and *β-*caryophyllene were observed higher in corolla oil and in aerial parts oil. Sixteen components (total 10.89%) in corolla and 17 components (total 7.73%) in aerial parts were found below 1%.

2*-β*-Pinene, 1-octen-3-ol, *α-*thujone, dihydrocarvone, thymol, *δ*-cadinene, and 1H-cycloprop[e]-azulen-7-ol were found only in the aerial parts essential oil. Also, *α-*phellandrene, linalool, linalyl butyrate, borneol, isoelemicin, *S*-indacene-1,7-dione, and 6-(2-formylhydrazino)-N,N ^'^–bis (isopropyl)-1,3,5-triazine-2,4-diamine were found only in the corolla essential oil. It was reported that linalool acetate, borneol and β-caryophyllene were found in amounts of 1.7%, 1.6% and 1.3%, respectively, in the essential oil isolated by hydrodistillation from the aerial parts of *O. acutidens* [8]*.* Also, borneol (1.5%-2.0%) and terpinene-4-ol (1.0% - 1.4%) were recorded in a previous study on essential oil of oregano [10]. In our study, borneol (1.19%) was found in the corolla oil, but this component was not detected in the aerial part oil. *β*-Caryophyllene and terpinene-4-ol were recorded in both corolla (2.04% and 0.62%, respectively) and in aerial parts (1.62% and 0.62%, respectively) essential oils.

The two essential oil samples obtained from aerial parts and corollas of *O. acutidens* were screened for antibiotic activity against ten bacterial strains. Table 2 and Table 3 summarize the antibacterial properties of the two essential oils, which exhibited a broad-spectrum of activity against both Gram-positive and Gram-negative bacteria. 

**Table 2:** Antibacterial activity against Gram-positive bacteria of the aerial parts and corolla essential oils from *O. acutidens.*

Mean diameter of inhibitory zones (mm±SE)^a^			
Treatments	*S. aureus*	*S. epidermidis*	*S. pyogenes*
Aerial Parts	56.0±0.0^b^	52.25±0.14^a^	82.5±1.44^a^
Corolla	60.0±0.0^a^	33.50±0.57^b^	85.0±0.7^a^
Chloramphenicol (30 µg)	26.00±0.57^c^	29.75±1.18^c^	33.75±1.31^d^
Tetracycline (30 µg)	31.50±0.28^d^	9.25±0.25^f^	43.75±2.39^bc^
Ampicillin (10 µg)	39.00±2.48^c^	19.00±1.00^e^	46.25±2.39^b^
Carbenicillin (100 µg)	40.50±0.28^c^	23.75±0.62^d^	43.00±2.64^bc^
Erythromycin (15 µg)	25.25±0.25^e^	32.50±2.25^bc^	38.25±1.65^cd^

^a ^The values are means ± standard error (n=4); Means with the same letter within columns are not significantly different at *p*>0.05; -:no activity.

**Table 3:** Antibacterial activity against Gram-negative bacteria of the aerial parts and corolla essential oils from *O. acutidens.*

Mean diameter of inhibitory zones (mm±SE)^a^							
Treatments	*S. marcescens*	*S. typhimurium*	*P. aeruginosa*	*P.vulgaris*	*K.pneumoniae*	*E.cloacae*	*E.coli*
Aerial Parts	10.5±0.28^e^	26.0±0.57^bc^	7.0±0.0^bc^	33.5±0.28^a^	14.5±0.28^c^	24.0±0.57^e^	13.5±0.28^c^
Corolla	18.0±0.0^c^	33.0±0.0^a^	9.0±0.0^b^	31.0±0.57^a^	20.5±0.28^b^	25.0±0.0^de^	22.75±0.28^b^
Chloramphenicol (30 µg)	27.75±0.62^a^	27.75±1.31^b^	10.75±1.49^b^	20.50±1.25^b^	28.50±0.95^a^	30.50±0.64^ab^	27.25±0.85^a^
Tetracycline (30 µg)	23.00±0.91^b^	26.25±1.31^bc^	18.50±1.32^a^	30.75±1.88^a^	27.75±1.31^a^	28.75±1.03^bc^	29.00±0.70^a^
Ampicillin (10 µg)	14.25±1.25^d^	26.25±0.75^bc^	- -	21.00±2.38^b^	- -	27.00±1.08^cd^	20.75±0.47^b^
Carbenicillin (100 µg)	25.25±0.47^b^	24.00±1.29^c^	23.25±2.49^a^	31.75±1.65^a^	- -	32.50±1.70^a^	22.75±1.03^b^
Erythromycin (15 µg)	10.75±1.18^e^	11.50±0.28^d^	18.50±5.54^a^	11.00±0.57^c^	12.75±0.85^c^	- -	15.25±2.13^c^

^a ^The values are means ± standard error (n=4); Means with the same letter within columns are not significantly different at *p*>0.05; -:no activity

The phenolic components such as thymol, carvacrol, carvacrol methyl ether and *p*-cymene are mainly responsible for the antibacterial properties of essential oils [11]. The principal active component of *Origanum* essential oils is carvacrol [3,4] and, for instance, *Origanum minutiflorum* carvacrol-rich oil (73.9%) exhibited strong antimicrobial activity [12]. Likewise, *Origanum scabrun*, which contains 75% of carvacrol, had a very high antibacterial effect against *S. aureus* and *E. coli* [4]. It was also stated that thymol and carvacrol were the main components of *Origanum glandulosum* essential oil and, also that the high level of thymol contained in *O. glandulosum* oil is responsible for the observed strong biological activity [5]. *p*-Cymene, which is the biological precursor of carvacrol, is not an effective antibacterial when used alone [13,14], but when combined with carvacrol, a synergistic effect against *B. cereus* has been reported [13].

**Figure 2:**
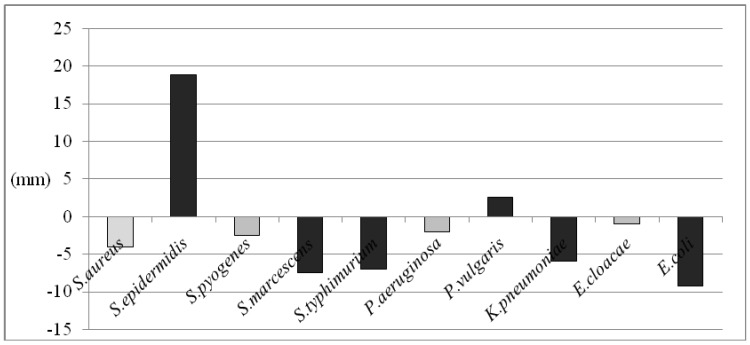
Inhibition zone differences between the aerial parts and corolla essential oil with respect to used bacteria strains (mm).

The aerial parts and corolla essential oils gave the best inhibitory activity against *Streptococcus pyogenes*, *Staphylococcus aureus* and *Staphylococcus epidermidis*, which are Gram-positive bacteria. In our study, inhibition zones of the essential oil from aerial parts and corolla varied from 82.5 and 85.0 mm to 52.3 and 33.5 mm, respectively, for the Gram-positive bacteria used. Although the two essential oil samples exhibited similar growth inhibitory activity against *S. pyogenes* and *S. aureus,* the aerial part essential oil was more active against *S .epidermidis* than the corolla oil. As to the Gram-negative bacteria, inhibition zones of the essential oil from aerial parts and corollas varied from 33.5 and 33.0 mm to 7.0 and 9.0 mm, respectively. The susceptibility of these bacteria changed depending on the essential oil sample investigated. Our findings showed the following order in the sensitivity to the essential oils, measured by the respective inhibition zones, *P. vulgaris >P. typhimurium > E. cloacae >, K. pneumonia >E. coli >S. marcescens >P. aeruginosa* for the aerial parts essential oil, and *P. typhimurium >P. vulgaris >E. cloacae >E. coli > K .pneumoniae >S. marcescens >P. aeruginosa* for the corolla essential oil (Figure 2). 

On the whole, the Gram-positive bacteria used in our study were more susceptible against the essential oil samples. As known, the Gram-positive bacteria seem to be more susceptible to the inhibitory effects of the essential oil than the Gram-negative bacteria, a feature attributed to the differences in the respective cell wall structures. The greater susceptibility of the Gram-positive bacteria may result from their simpler single layer cell wall structures, while the Gram-negative cell wall is a multi-layered structure and quite complex [15,16]. It has been reported that the phenolic monoterpene carvacrol is able to disintegrate the outer membrane of Gram-negative bacteria, releasing lipopolysaccharides (LPS) and increasing the permeability of the cytoplasmic membrane to adenosine triphosphate (ATP) [16,17]. This was shown to damage the cells irreversibly. Our findings are in agreement with these data. On the other hand, it was reported that essential oils extracted by hydrodistillation from the aerial parts of 23 samples of Algerian *Origanum glandulosum* Desf. were characterized by the predominance of four components: thymol (18.5–73.1%), carvacrol (7.6–72.6%), *p*-cymene (1.7–18.5%) and γ-terpinene (1.1–18.7%), and all microbial strains (*Escherichia coli*, *Pseudomonas aeruginosa*, *Staphylococcus aureus*, *Enterococcus hirae*, *Candida albicans*, *Candida tropicalis*) tested (Gram-positive and Gram-negative bacteria and yeasts) showed a fairly similar degree of susceptibility to the essential oils under investigation, although no evident difference was observed in their sensitivity [18]. Similarly, the essential oils of *O. vulgare* subsp. *hirtum* appeared to be equality effective against both Gram-positive and Gram-negative microorganisms [17].

Positive controls (reference antibiotics) showed antibacterial activity towards the Gram-positive bacteria used, but no inhibition by ampicillin against *P. aeruginosa* and *K. pneumoniae*, carbenicillin against *K. pneumoniae*, and erthromycin against *E. cloacae* were observed. In addition, the essential oil from flowers showed stronger antibacterial efficacy against all the microorganisms, except *S. epidermidis* and *P. vulgaris*, used in our study (Table 2 and Table 3). 

Twenty-three minor components, constituting 24.04% and 18.17% of essential oil obtained from *O. acutidens* corolla and aerial parts, respectively were detected (Table 1). There is some evidence that minor components also have a critical part to play in antibacterial activity, possibly by producing a synergistic effect between other components [19]. This has been found to be the case for sage [20], certain species of *Thymus* [21,22] and oregano [21].

When the our results were compared with the literature, the chemical composition and content of essential oil from *O. acutidens*, similarly other medicinal plants, in the present study showed significant differences, which can be attributed several factors, such as the part of plant under analysis, the stage of plant development, the time of harvesting or picking, differences in climatic and ecological conditions, and the different distillation methods used in the studies etc. [15,16,17,18,19,20,21,22,23] and the antimicrobial activities of plants depends on the type, composition and concentration of essential oils. Essential oils rich in phenolic compounds, such as carvacrol, are widely reported to possess high levels of antimicrobial activity [4,24].

On the whole, this study demonstrates the antibacterial activity of the essential oil of this endemic species against the tested bacterial strains, being the first report on the components and antibacterial activity of the corolla essential oil of *O. acutidens*. In view of the obtained inhibitory responds of its aerial parts and corolla, it is suggest that these essential oils or the natural components found in the essential oil of *O. acutidens* could be used in the food and pharmaceutical industries, and as an alternative to common synthetic antimicrobial products.

## Experimental

### Plant Material

This study was carried out at the laboratories of the Field Crops Department, Faculty of Agriculture of Ankara University. The herbal parts of *Origanum acutidens* (Hand.-Mazz.) Ietswaart grown at the experimental field of this Department were collected when flowering (July 30, 2007). The collected plants were dried in the shade at room temperature. 

### Isolation of essential oil

Dried aerial parts (leaves and stems) and corolla parts of the flowers (about 50 g) were ground and subjected to hydrodistillation for 3 h in 500 mL water, using a Clevenger-type apparatus. 

### Gas chromatographic-mass spectrometric analysis of essential oil

All gas chromatography (GC) analyses were carried out on a Hewlett Packard 6890 N GC instrument, fitted with a HP 5MS 30 m×0.25 mm×0.25 μm film thickness capillary column and FID detector. The column temperature was programmed from 50°C to 150°C at an initial rate of 3°C/min. The injector and detector temperatures were programmed at 220°C and 290°C, respectively. Helium was used as the carrier gas at a flow rate 1 mL/min. The gas chromatography-mass spectrometry (GC/MS) analyses were performed using a Hewlett Packard 5973 (mass selective detector)-6890 GC/MS system operating in the electron ionization system with ionization energy of 70 eV (equipped with a HP 5MS 30 m × 0.25 mm × 0.25 μm film thickness capillary column), using He (1 mL/min) as the carrier gas. The initial temperature of the column was 50°C and then heated gradually to 150°C with a 3°C/min rate, held for 10 min and finally raised to 250 ^◦^C/min. Diluted samples (1/100 in acetone, v/v) of 1.0 µL were injected automatically and in the splitless mode. The identification of chemical compounds obtained from our study was performed by matching their retention times and mass spectra with those obtained from the Flavor2.L, Wiley7n.1 and NIST98.L spectral and literature data [25]. Relative percentages of the separated compounds were calculated from FID chromatograms.

### Antibacterial bioassay

The disc diffusion assay (Kirby-Bauer Method) was used to screen for antibiotic activity [26]. The microorganisms used were: *Escherichia coli* (ATCC^®^25922), *Pseudomonas aeruginosa* (ATCC^®^27853), *Salmonella typhimurium* (ATCC^®^14028), *Serratia marcescens* (ATCC^®^8100), *Proteus vulgaris* (ATCC^®^13315), *Enterobacter cloacae* (ATCC^®^23355) and *Klebsiella pneumoniae* (ATCC^®^13883) which are Gram-negative bacteria and *Streptococcus pyogenes* (ATCC^®^19615), *Staphylococcus aureus* (ATTC^®^25923) and *Staphylococcus epidermidis* (ATCC^®^12228) which are Gram-positive bacteria. The pathogenic organisms were selected for the study on the basis of their clinical and pharmaceutical importance. 

BD-Microtrol discs (Becton Dickinson Laboratories, France) containing different bacterial strains were transferred to test tubes containing 2 mL of Tryptic Soy Broth (TSB) and incubated for 3 hours at 37°C. After 3 hours, one bacteriological loop from each broth was streaked on Tryptic Soy Agar (TSA) plates and incubated for 2 days at 37°C. After 2 days, 4-5 loops of pure culture were transferred to 20 mL of TSB in a test tube for each bacterial strain and incubated overnight at 37°C; then 0.2 mL from each culture was transferred into 0.8 mL TSB in eppendorf tubes. A sterile cotton swab dipped into the bacterial suspension and 0.L mL from each strand was transferred into Mueller-Hinton (M-H) agar plate (One Eppendorf tube was used for each agar plate). The plates were inoculated with a microorganism suspension at a density of 10^6^ cells/mL. Agar plates were streaked four times, each time turning the plate at a 90° angle and finally rubbing the swab through the edge of the plate. All extracts were sterilized by filtering through a 0.22 µm filter (Pal-Gelman Laboratory) and sterile paper discs (Glass Microfibre filters, Whatman^®^; 6 mm in diameter) were soaked in the extract and placed onto inoculated plates. The inoculum for each bacterium was prepared from broth cultures. The amounts of the undiluted essential oils applied were 10 μL. There were four replicates for each extract tested for each bacterium. Positive controls consisted of five different antimicrobial susceptibility test discs (Bioanalyse^®^): erythromycin (15 µg) (E-15), ampicillin (10 µg) (AM-10), carbenicillin (100 µg) (CB-100), tetracycline (30µg) (TE-30) and chloramphenicol (30 µg) (C-30). One antibiotic disc was used for each plate and run in duplicate. Inoculated plates with discs were placed in a 37°C incubator. After 16 to 18 hours of incubation, the results were recorded by measuring the zones of growth inhibition surrounding the disc. Clear inhibition zones around the discs indicated the presence of antimicrobial activity. 

### Statistical analysis

The results obtained from antibacterial bioassays were expressed as means ± standard error of the mean. All data were analyzed by analysis of variance (ANOVA) and mean values were compared with Duncan’s Multiple Range Tests using SPSS vers. 15 (SPSS Inc., Chicago, IL, USA) [27]. 

## References

[1] Vural M. (2003). Türkiye’nin Tehlike Altındaki Bitkileri. Türkiye’de Biyolojik Çeşitlilik ve Organik Tarım Çalıştay Raporu.

[2] Tumen G., Baser K.H.C., Kirimer N., Ozek T. (1995). Essential oil of *Origanum saccatum* P.H. Davis. J. Essent. Oil Res..

[3] Sivropoulou A., Papanikolaou E., Nikolaou C., Kokkini S., Lanaras T., Arsenakis M. (1996). Antimicrobial and cytotoxic activities of *Origanum* essential oil. J. Agric. Food Chem..

[4] Aligiannis N., Kalpoutzakis E., Mitaku S., Chinou I.B. (2001). Composition and antimicrobial activity of the essential oils of two *Origanum species*. J. Agric. Food Chem..

[5] Bendahou A., Muselli A., Grignon-Dubois M., Benyoucef M., Desjobert J.M., Bernardini A.F., Costa J. (2008). Antimicrobial activity and chemical composition of *Origanum glandulosum* Desf. essential oil and extract obtained by microwave extraction: Comparison with hydrodistillation. Food Chem..

[6] Baser K.H.C., Tumen G., Duman H. (1997). Essential oil of *Origanum acutidens* (Hund. Murr.) lestwaart. J. Essent. Oil Res..

[7] Sokmen M., Serkedjieva J., Daferera D., Gulluce M., Polissiou M., Tepe B., Akpulat H. A., Sahin F., Sokmen A. (2004). In vitro antioxidant, antimicrobial and antiviral activities of the essential ail and various extracts from herbal parts and callus cultures of *Origanum acutidens*. J. Agric. Food Chem..

[8] Kordali S., Cakir A., Ozer H., Cakmakci R., Kesdek M., Mete E. (2008). Antifungal, phytotoxic and insecticidal properties of essential oil isolated from Turkish *Origanum acutidens* and its three components, carvacrol, thymol and p-cymene. Bioresour.Technol..

[9] Baser K.H.C. (2002). Aromatic biodiversity among the flowering plant taxa of Turkey. Pure Appl. Chem..

[10] Figuérédo G., Chalchat J.C., Pasquier B. (2006). Studies of Mediterranean oregano populations IX: Chemical composition of essential oils of seven species of oregano of various origins. J.Essent. Oil Res..

[11] Cosentino S., Tuberoso C.I.G., Pisano B., Satta M., Mascia V., Arredi E., Palmas F. (1999). In vitro antimicrobial activity and chemical composition of Sardinian *Thymus* essential oils. Lett. Appl. Microbiol..

[12] Aslim B., Yucel N. (2008). In vitro antimicrobial activity of essential oil from endemic *Origanum minutiflorum* on ciprofloxacin-resistant *Campylobacte*r spp. Food Chem..

[13] Ultee A., Bennik M.H.J., Moezelaar R. (2002). The phenolic hydroxyl group of carvacrol is essential for action against the food-borne pathogen *Bacillus cereus*. Appl. Environ. Microb..

[14] Burt S. (2004). Essential oils: their antibacterial properties and potential applications in foods-a review of the literature. Int. J. Food Microbiol..

[15] Esen G., Azaz A.D., Kurkcuoglu B., Baser K.H.C., Tınmaz A. (2007). Essential oil and antimicrobial activity of wild and cultivated *Origanum vulgare* L. subsp. *hirtum* (Link) letswaart from the Marmara region, Turkey. Flav. Fragr. J..

[16] Turker A.U., Usta C. (2008). Biological screening of some Turkish medicinal plant extracts for antimicrobial and toxicity activities. Nat. Prod. Res..

[17] Dorman H.J.D., Deans S.G. (2000). Antimicrobial agents from plants: antibacterial activity of plant volatile oils. J. Appl. Microbiol..

[18] Sari M., Biondi D.M., Kaâbeche M., Mandalari G., D’Arrigo M., Bisignano G., Saija A., Daquino C., Ruberto G. (2006). Chemical composition, antimicrobial and antioxidant activities of the essential oil of several populations of Algerian *Origanum glandulosum* Desf. Flav. Fragr. J..

[19] Unlu G.V., Unlu M., Donmez E., Vural N. (2007). Chemical composition and in vitro antimicrobial activity of the essential oil of *Origanum minutiflorum* O Schwarz & PH Davis. J. Sci. Food Agric..

[20] Marino M., Bersani C., Comi G. (2001). Impedance measurements to study the antimicrobial activity of essential oils from *Lamiaceae* and *Compositae*. Int. J. Food Microbiol..

[21] Paster N., Menasherov M., Ravid U., Juven B. (1995). Antifungal activity of oregano and thyme essential oils applied as fumigants against fungi attacking stored grain. J. Food Prot..

[22] Marino M., Bersani C., Comi G. (1999). Antimicrobial activity of the essential oils of *Thymus vulgaris L*. measured using a bioimpedometric method. J. Food Prot..

[23] Milos M., Mastelic J., Jerkovic I. (2000). Chemical composition and antioxidant effect of glycosidically bound volatile compounds from Oregano (*Origanum vulgare* L. ssp. *hirtum*). Food Chem..

[24] Baydar H., Sagdic O., Ozkan G., Karadogan T. (2004). Antibacterial activity and composition of essential oils from *Origanum*, *Thymbra* and *Satureja* species with commercial importance in Turkey. Food Contr..

[25] Sahin F., Gulluce M., Dafera D., Sokmen A., Sokmen M., Polssiou M., Agar G., Ozer H. (2004). Biological activities of the essential oils and methanol extract of *Origanum*
*vulgare* ssp. *vulgare* in the Eastern Anatolia region of Turkey. Food Contr..

[26] Prescott L.M., Harley J.P., Klein D.A. (1990). Microbiology Antimicrobial Chemotherapy.

[27] Kalaycı S. (2008). SPSS Uygulamalı Cok Degiskenli Istatistik Teknikleri.

